# Serological and Molecular Investigation of *Coxiella burnetii* in Small Ruminants and Ticks in Punjab, Pakistan

**DOI:** 10.3390/ijerph16214271

**Published:** 2019-11-04

**Authors:** Qudrat Ullah, Hosny El-Adawy, Tariq Jamil, Huma Jamil, Zafar Iqbal Qureshi, Muhammad Saqib, Shakeeb Ullah, Muhammad Kamal Shah, Alam Zeb Khan, Muhammad Zubair, Iahtasham Khan, Katja Mertens-Scholz, Klaus Henning, Heinrich Neubauer

**Affiliations:** 1 Faculty of Veterinary and Animal Sciences, Gomal University, Dera Ismail Khan 29050, Pakistan; qudratmahsud@gmail.com (Q.U.); shakeebullah@gmail.com (S.U.); kamaluaf560@gmail.com (M.K.S.); dralamzebkhan2010@hotmail.com (A.Z.K.); 2 Department of Theriogenology, University of Agriculture Faisalabad, Faisalabad 38000, Pakistan; drhjamil@hotmail.com (H.J.); zafaruaf2013@gmail.com (Z.I.Q.); 3 Friedrich-Loeffler-Institut, Institute of Bacterial Infections and Zoonoses, 07743 Jena, Germany; tariq.jamil@fli.de (T.J.); Katja.Mertens-Scholz@fli.de (K.M.-S.); Klaus.Henning@fli.de (K.H.); heinrich.neubauer@fli.de (H.N.); 4 Faculty of Veterinary Medicine, Kafrelsheik University, Kafr El-Sheik 35516, Egypt; 5 Department of Clinical Medicine and Surgery, University of Agriculture Faisalabad, Faisalabad 38000, Pakistan; drsaqib_vet@hotmail.com; 6 Faculty of Veterinary and Animal Sciences, University of Poonch Rawalakot, Azad Kashmir 12350, Pakistan; drzubairabbasi@gmail.com; 7 Section of Epidemiology and Public Health, University of Veterinary and Animal Sciences, Lahore, sub-campus Jhang, Jhang 35200, Pakistan; iahtasham.khan@uvas.edu.pk

**Keywords:** *Coxiella burnetii*, Q Fever, small ruminants, ELISA, PCR, ticks

## Abstract

Coxiellosis is a zoonotic disease caused by the obligate intracellular bacterium *Coxiella burnetii* affecting the productive and reproductive capabilities of animals. This study was conducted to gain insight into the seroprevalence of coxiellosis in small ruminants in seven farms of the Punjab, Pakistan. Potential risk factors were assessed. In total, 1000 serum samples (500 from sheep and 500 from goats) and 163 ticks were collected from the ruminants. All these 163 ticks were merged into 55 pools (29 pools for ticks from sheep and 26 pools for ticks from goat). Serum samples were investigated using an indirect ELISA and PCR. *Coxiella burnetii* DNA was detected in 29 pooled seropositive samples and 11 pooled ticks by real-time qPCR. Serological analysis revealed a prevalence of 15.6% and 15.0% in sheep and goats, respectively. A significant association was found between seropositivity and different variables like district, lactational status, reproductive status, body condition and reproductive disorders. Univariate analysis showed that detection of *C. burnetii* DNA in tick pools was significantly associated with the presence of ticks on sheep and goats. However, a non-significant association was found for the prevalence of *C. burnetii* DNA in serum pools. Hence, *C. burnetii* infection is prevalent in small ruminants and ticks maintained at livestock farms in Punjab, Pakistan.

## 1. Introduction

Coxiellosis (Q Fever) is a zoonotic bacterial infection affecting various hosts including humans, ruminants, and pets. The pathogenic agent is to be found everywhere with the exception of New Zealand and French Polynesia [1,2]. Coxiellosis is caused by *Coxiella* (*C*.) *burnetii*, an obligate Gram-negative intracellular bacterium [3,4]. *Coxiella burnetii* can propagate within the acidic phagolysosomal vacuole of mononuclear phagocytes and shows two distinct life cycles and lipopolysaccharide (LPS) phase variation between phase I and phase II [5]. *Coxiella burnetii* was detected in all animal species and humans but small ruminants are the most important reservoir and common source of human infection [6]. The largest Dutch Q fever outbreak (2007–2010) with more than 4000 reported human cases was primarily linked to dairy goat farms [7]. The bacteria are mainly transmitted by aerosols. Humans usually get Q fever by breathing in contaminated droplets released by birth products of infected animals and drinking raw milk. Coxiellosis may occasionally be transmitted vertically or sexually but these are not common routes. Ticks may play a role in the transmission of disease in animals but this is questioned for human infection [6,8].

Coxiellosis is usually asymptomatic with sub-clinical presentation in animals and usually not considered a problem for animal health except in ruminants where it causes reproductive problems like abortion, premature delivery, stillbirth and weak offspring [9,10,11]. Abortion is the most important clinical manifestation of coxiellosis in sheep and goats with an incidence of 3% to 80% [12]. In cattle, infertility is the main clinical manifestation [13].

Different techniques can be used for the diagnosis of *C. burnetii* infection in animals but ELISA for serological investigation and PCR for molecular detection of *Coxiella* DNA are believed to be the most accurate ways to diagnose [8,14].

In Pakistan, coxiellosis (Q fever) is one of the highly neglected diseases in humans and animal species. From 1955 to 2016, there are only six studies reported the prevalence of *C. burnetii* infection in humans and animals and most of them are based on a complement fixation test (CFT). According to these studies, the prevalence of coxiellosis ranges from 4.6% to 40% in all livestock species and 10.2% to 26.8% in humans [8,15,16,17,18,19]. In view of the steady increase of the prevalence of this disease worldwide, the present study was designed to investigate the prevalence of coxiellosis and its association with potential associated risk factors in small ruminants maintained at livestock farms of Punjab, Pakistan.

## 2. Materials and Methods

### 2.1. Description of Study Area

This study was carried out in small ruminants kept in seven different livestock farms of the Punjab province of Pakistan. Punjab is the largest province, with the highest human and animal population in Pakistan. It is also the second largest province in the country, with an area of 205,344 km^2^. Geographically, it is located at 31.1704°N and 72.7097°E in the semiarid lowland region. The temperature ranges from −2 to 45 °C but can reach 50 °C (122 °F) in summer and may fall to −10 °C in winter. Mean annual rainfall varies greatly with the highest rainfall in the northern regions [8,18,20]. Agriculture and livestock are the major sources of socio-economic development especially in rural regions of Pakistan. As per the livestock census of Pakistan, Punjab dominates the livestock population with 49%, 65%, 24% and 37% of the cattle, buffaloes, sheep and goats of the country, respectively [21].

Since, no previous studies on *C. burnetii* have been conducted in these districts so far, it might help us to select a particular region/area to be studied. We choose districts, which reflect the major livestock population in the province with an increased annual incidence of livestock-associated zoonotic diseases (Directorates of Animal and Human Health, Punjab).

### 2.2. Estimation of Sample Size

This study was conducted during January to June 2016 in seven governmental livestock farms located in the Khanewal, Khushab, Rajanpur, Bhawalpur, Layyah, Okara and Bhakkar districts of Punjab province, Pakistan. Survey Toolbox software (Ausvet, The Australian Biosecurity Cooperative Research Centre for Emerging Infectious Disease, Australia) was used for the random selection of farms and animals [22]. The prevalence of Q fever in small ruminants in the study areas was unknown. For the calculation of sample size, a 95% confidence interval with an expected prevalence of 50% and desired absolute precision of 5% was applied [23]. Three hundred and eighty-four serum samples were required from all farms. In total, 1000 samples (500 from sheep and 500 from goats) were collected. During sampling, data on the individual animal, i.e., species, locality of farm (districts), age, sex, parity, lactational status, reproductive status (pregnant or non-pregnant), body condition, and reproductive disorders, and general farm management were recorded using a structured questionnaire. 

### 2.3. Sample Collection 

Approximately 10 mL of blood was collected from the jugular vein of each animal using disposable needles and evacuated blood collection tubes (Improvacuter, Shanghai International Hamburg Holding, GmbH, Germany). Each tube was properly labeled for its identification and information about its origin. Blood collection tubes were placed vertically in a cool box packed with gel freezing pads and shipped to the laboratory for further processing. At the laboratory, centrifugation was performed at 4500 rpm for 10 min for proper separation of the serum from whole blood. The serum was preserved in disposable screw-capped cryovials (Cryo.S^TM^; Greiner Bio-one, GmbH Frickenhausen, Germany) and stored at −20 °C until use for further investigation.

### 2.4. Collection and Handling of Ticks 

During the serum sampling (January to June, 2016), 163 ticks (85 from sheep and 78 from goats) were collected from the same seven governmental livestock farms where serum samples were collected. Out of 163 ticks, 85 ticks were collected from sheep, while 78 were collected from goats. The ticks were placed in properly labeled bottles containing 70% ethyl alcohol as a preservative at the University of Agriculture Faisalabad. Ticks were shipped to Friedrich-Loeffler-Institut (FLI), Jena, Germany, following international regulations for transportation. Ticks were merged into 55 pools (29 pools from sheep and 26 pools from goats). Each pool was composed of 3 ticks (with only pool no. 29 containing 1 tick). These pools were then stored at −20 °C until use for the detection of *Coxiella* DNA.

### 2.5. Ethics Statement

Blood samples were collected from small ruminants as per the guidelines of the International Animal Care and Use Committee (IACUC) and after obtaining the consent of the Secretary, Livestock and Dairy Development department, Punjab, Pakistan (wide letter No.SO (I&C)/L7DD/2-6/2016). The samples were processed based on the approval of the Ethical Research Board at the University of Agriculture Faisalabad, Pakistan (wide letter No. ORIC/3253, dated November 16, 2013). 

### 2.6. Diagnostic Tests

#### 2.6.1. Indirect ELISA

All serum samples were analyzed by Indirect ELISA (IDEXX Q-Fever (*Coxiella burnetii*) Antibody Test Kit, Germany). The plates were read at 450 nm with an ELISA reader (Anthos 2020, Wals, Austria) according to the manufacturer’s recommendation. To calculate the results, the optical densities (ODs) of the sera were considered to be ELISA-positive if they had an OD value of 40% or more, suspected if the value was between 30% and 40%, and were considered negative if the OD value was <30%. Confirmed positive results by indirect ELISA were considered as a final criteria for the calculation of seroprevalence.

#### 2.6.2. Real-Time qPCR

After serological analysis, 29 pools of 153 seropositive and 81 suspected serum samples and 10 pools of seronegative samples were analyzed using TaqMan-based real-time PCR assay targeting the multi copy *IS1111* element as well as the single copy *icd* gene of *C. burnetii* [24]. Each serum pool was comprised of six serum samples. All molecular procedures were carried out in specially designed cabins to prevent any kind of contamination. DNA was extracted using a high pure PCR template preparation kit (Roche kit, version 20, Roche Diagnostics GmbH, Germany).

The surface of ticks was washed by serial passages in 10% and/or 70% ethyl alcohol and rinsed in sterile water. DNA was extracted from 55 tick pools as described by using the protocol for the purification of genomic DNA from insects using high pure PCR template preparation kit (Roche, Roche Diagnostics GmbH, Germany) and investigated using real-time qPCR targeting multiple copy *IS1111* transposase gene as well as the *icd* gene of *C. burnetii*. Real-time qPCR performed in duplicate.

In each qPCR run, plasmid standards (for *IS1111* from 10^5^ to 10^1^, while for *icd* 10^6^ to 10^1^) were used as a positive control and DNase and RNase-free water was used as a negative control. Results of qPCR were counted as positive at a cut off ≤35. A standard curve was generated using decimal dilutions of the positive control. The PCR primer sequences and conditions were applied as described by Chakrabartty et al. 2016 [25].

### 2.7. Statistical Analysis

Minitab17 software (Minitab, LLC, State College, PA, USA) was used for calculation of prevalence percentage and respective 95% confidence intervals. ODD ratios, respective 95% confidence interval and Chi-square values were calculated using IBM SPSS Statistics 13.0 for Windows^®^ (IBM Corporation, Armonk, NY, USA). Chi-square test was applied for the calculation of the significance of association (*p* < 0.05) between the seroprevalence of coxiellosis and various farm-related variables.

## 3. Results 

### 3.1. Indirect ELISA Results

The results of iELISA showed that 153 serum samples were seropositive, and 81 serum samples were considered as suspected. Univariate analysis was conducted to investigate the relationship of seven livestock farms and associated variables with the prevalence of *C. burnetii* infection (Table 1 and Table 2). The overall prevalence of coxiellosis in seven districts of the Punjab province was found to be 15.3%. The highest prevalence (26.2%) was recorded in the district Layyah, while the lowest prevalence (5.8%) was reported in animals of the Rajanpur and Khanewal district. Statistical analysis showed a significant (*p* = 0.000) difference for the prevalence in various districts. There is no significant difference between seroprevalence in sheep (15.6%) and goats (15.0%). Male animals showed higher seroprevalence (18.7%) than females (14.9%) (*p* = 0.302). However, seropositivity for *C. burnetii* infection was non-significantly associated with age (*p* = 0.085) and parity (*p* = 0.838). A higher significant prevalence (20.0%) was recorded in non-lactating animals than in lactating animals (8.5%) (*p* = 0.000). Regarding the reproductive status, the anti-*Coxiella* antibodies were detected in 17.3% and 10.6% of non-pregnant and pregnant animals, respectively (*p* = 0.002). 

The prevalence of *C. burnetii* infection in animals with a history of various reproductive problems was significantly different (*p* = 0.000). The seroprevalence in animals with a previous history of abortion, premature delivery, stillbirth and repeated breeding was 51.6%, 32.4%, 31.8% and 25%, respectively.

As far as body condition was concerned, higher prevalence, (44.9%) was recorded in small ruminants with weak body condition, while lower prevalence (37.7%) was found in ewes with the delivery of a weak lamb. Statistical analysis revealed that the prevalence of the disease was significantly different (*p* = 0.000) between weak animals and animals with a weak lamb or kid delivery. 

### 3.2. Real-Time qPCR Results

#### 3.2.1. Serum Pools

Out of 29 serum pools tested with qPCR, *C. burnetii* DNA was not detected in seropositive or seronegative pools. *Coxiella burnetii* DNA was amplified in five (17.2%) serologically suspected serum pools. Statistical analysis revealed that the prevalence of *C. burnetii* DNA in serum pools was not significantly (*p* = 0.333) different between sheep (13.3%) and goats (21.4%) (Table 3). 

#### 3.2.2. Ticks Pools

*Coxiella burnetii* DNA was detected in 20% of tick pools. Univariate analysis of data revealed a significant difference in the prevalence of *C. burnetii* DNA in tick pools collected from sheep (31.0%) and goats (7.7%) (*p* = 0.031, df = 1, χ^2^ = 4.668) (Table 4). 

## 4. Discussion

Coxiellosis is a zoonotic disease caused by *C. burnetii*. The diagnosis of *C. burnetii* infection in animals is of great importance not only to identify the infected flocks but also to determine the risk of disease transmission to humans [8,26]. The epidemiological information about the geographic distribution of *C. burnetii* infection is very limited in humans and livestock populations in Pakistan. To the best of our knowledge, this is the first epidemiological survey investigating the prevalence of coxiellosis in small ruminants and ticks maintained at different governmental livestock farms in Punjab, Pakistan. In the current study, the prevalence of *C. burnetii* infection in small ruminants has been investigated using ELISA and real-time qPCR. While in ticks, real-time PCR assay was used for the detection of *C. burnetii* DNA in pooled samples.

In this study, the prevalence of coxiellosis varied greatly in the seven districts of the Punjab province. The prevalence ranged from 26.2% (95% CI: 21.1–31.7) in district Layyah to 5.8% (95% CI: 3.2–9.5) in districts Rajanpur and Khanewal. Univariate analysis proved a significant (*p* = 0.000) association of prevalence and district. Ezatkhah et al. (2015) recorded a seroprevalence of 26.4% in small ruminants of five counties of the southeast region of Iran, which ranged from 5% in Sarbaz to 39.2% in Iranshahr [27]. The higher prevalence of the disease might be due to the prevailing climatic and weather conditions in the region studied [28]. This variation in the prevalence of infection within flock and different geographical areas might be associated with the farm hygienic measures, routine management practices and environmental factors such as vegetation, soil moisture and the presence of infected animals in the surroundings [7,29]. These management and environmental factors might be responsible for the higher seroprevalence of coxiellosis in different districts. A significant high prevalence of 69.4% and 75.0% was reported previously by Zahid et al. (2016) in the Layyah and Muzaffargarh districts of Punjab province, respectively [19]. On the other hand, a very low seroprevalence (5.8%) was recorded in the districts Rajanpur and Khanewal, which might be attributed to better hygienic practices.

Results of this study revealed an overall seroprevalence in small ruminants of 15.3% (95% CI: 12.3–18.7), which varied from 15.6% (95% CI: 12.07–18.33) to 15.0% (95% CI: 12.62–18.98) between sheep and goats (*p* = 0.792). Rizzo et al., 2016, reported a similar flock level seroprevalence (14.5%) in sheep and goats [7]. However, a few previous studies reported higher seropositivity in sheep [30,31]. Controversially, other studies found higher prevalence in goats [7,32]. Variations in intrinsic susceptibility to this pathogen among small ruminant species have not been described in detail [32]. Thus, the species-wise variations in the prevalence of disease may be more likely attributed to different farm management practices and variation in flock density in sheep and goat farms [19,33]. This survey revealed that small ruminants are important reservoirs of *C. burnetii* infection in the study area.

Male animals showed a higher seroprevalence (18.7%) than females (14.9%) (*p* = 0.302). However, seropositivity for *C. burnetii* infection was non-significantly associated with age (*p* = 0.085) and parity (*p* = 0.838).

In the current study, the prevalence of *C. burnetii* infection varied greatly between non-lactating (20.0%) and lactating animals (8.5%) (*p* = 0.000). While in Kenya, the seroprevalence of coxiellosis was higher in lactating animals as compared to non-lactating animals, although the difference was not statistically significant [28]. Paul et al. (2012) reported a significantly (*p* < 0.005) higher prevalence of coxiellosis in dairy breeds as compared to the beef breeds of cattle [29]. The target sites for proliferation of *C. burnetii* are the placenta and mammary glands of animals [34,35]. In ruminants, immediately after inoculation, the organism reaches the predilection site through the blood stream and resides in the supramammary lymph nodes, mammary glands and placenta of pregnant animals, where it replicates and may contribute to higher prevalence in lactating animals [14].

In this study, the non-pregnant small ruminants showed higher seroprevalence (17.3%) than pregnant (10.6%) (*p* = 0.002), which is in agreement with the study conducted by Abushahba et al. (2017), which observed a non-significant difference regarding reproductive status in small ruminants and the prevalence of Q fever in Egypt [36]. The results of this study were in contrast to a study conducted previously in the Netherlands that reported a higher seroprevalence of *Coxiella* in pregnant and periparturient small ruminants when compared with non-pregnant ruminants and those in the early gestation period [37].

In this study, the prevalence of coxiellosis was higher (11.1%) in young non-pregnant animals aged from 1 to 2.5 years than in pregnant animals (6.8%). In middle-aged small ruminants (2.5 to 4 years), the prevalence was 19.7% and 16% in non-pregnant and pregnant, respectively. The older non-pregnant animals (≥4 years) showed a higher prevalence (17.5%) than pregnant animals of the same age (9.6%).

Interestingly, the prevalence of anti-Coxiella antibodies was 20% in young females (≤1 year). 

Results of the current study revealed that seroprevalence of coxiellosis was significantly (*p* = 0.000) different in animals with a previous history of reproductive problems including abortion (51.6%), premature delivery (32.4%), stillbirth (31.8%) and repeated breeding (25%). These findings are in agreement with the results of García-Pérez et al. (2009), who observed a significantly higher prevalence of coxiellosis in animals with a history of reproductive problems [38]. Controversially, Ruiz-Fons et al. (2010) and Asadi et al. (2013) did not record any association between the prevalence of coxiellosis and a history of reproductive disorders [39,40]. Antibodies against *C. burnetii* found in small ruminants with a history of reproductive problems do not necessarily mean that *C. burnetii* is the only cause of reproductive loss in these animals. Infection with other pathogens such as *Brucella melitensis* and *Toxoplasma gondii* may be possible confounders. Furthermore, malnutrition in pregnant females might be a reason for abortion. During the periparturient period, the massive multiplication of these pathogens occurs within trophoblast cells, causing necrotic suppurative placentitis, which ultimately leads to pregnancy failure in the form of abortion, stillbirth, premature delivery and birth of weak offspring [41,42].

A significant (*p* = 0.000, df = 3, χ^2^ = 124.868) difference in prevalence was found between *C. burnetii* seropositivity and animals with poor body conditions. Highest seropositivity was recorded in weak animals (45.7%) followed by the does that gave birth to weak kids (44.9%) and ewes that delivered weak lambs (37.7%). Agerholm (2013) also reported that *C. burnetii*-infected ewes gave birth to noticeably small and underweight lambs [43]. Similarly, Saegerman et al. (2013) found an association between *C. burnetii* seropositivity and delivery of weak calves (OR = 2.14 with 95% CI: 1.05–4.39) [44].

Infected goats with Q fever after abortion showed signs of endometritis. Full-term kids were weak, with low body weight and high mortality. Many of the apparently healthy kids infected with *C. burnetii* were suffering from digestive and respiratory tract problems [45]. 

The higher seropositivity in females with weak lamb and kid deliveries might be due to the massive bacterial multiplication that occurs during the periparturient period in pregnant animals [42]. 

Real-time PCR assay targeting a single copy of the *icd* gene was used for the detection of *C. burnetii* DNA [24]. Results of this study revealed that *C. burnetii* DNA was not detected in the seropositive samples. However, the DNA was detected in five (17.2%) pools of suspected serum samples. 

The presence of *C. burnetii* DNA in suspected serum pools indicated that infection was probably in the incubation period or in the early acute phase, as *C. burnetii* DNA cannot be detected in serum after two weeks of the onset of clinical infection [46,47,48,49].

Schneeberger et al. (2010) also recorded PCR positive results in 98% seronegative samples collected from acute Q fever patients [48]. Vincent et al. (2015) found that early serum samples from acute Q fever patients are infectious and an important source of viable *C. burnetii* [50]. Apart from serological investigation, *C. burnetii*-specific PCR can be effectively used for the genomic detection of coxiellosis in the early acute phase of Q fever, but contradictory sensitivities have also been reported [48,51]. Hence, qPCR can also be an additional tool to achieve a better understanding of the disease prevalence in the veterinary medicine practice.

*Coxiella burnetii* DNA was detected in 31.0% (95% CI: 15.3–50.8) and 7.7% (95% CI: 0.9–25.1) of ticks collected from sheep and goats, respectively. Univariate analysis revealed a significant (*p* = 0.031, df = 1, χ^2^ = 4.668) difference in the prevalence of *C. burnetii* DNA in tick pools collected from sheep and goats.

Knobel et al. (2013) showed that *C. burnetii* DNA can be detected in 2.5% and 20.0% of ticks collected from cattle and dogs, respectively [52]. In another study conducted in Ethiopia, a significant number of *C. burnetii* DNA (25.0%) was found in samples from questing ticks collected from domestic animals [53]. In contrast, Sprong et al. (2012) reported only 0.2% *C. burnetii* DNA in questing *Ixodes ricinus* ticks and ticks in wildlife, pets and domestic animals by using a multiplex q-PCR [54]. Although, some pioneering studies have focused on *C. burnetii* in ticks, the role of ticks in the epidemiology of Q fever remains uncertain. Even the highly virulent Nine Mile reference strain of *C. burnetii* was isolated from a guinea pig upon *Dermacentor andersoni* ticks had fed [55]. The occasional reports of an unexpectedly high prevalence of *C. burnetii* DNA in ticks may reflect their role as a vector for the transmission of Q fever [55].

The marker *IS1111* transposable element was routinely targeted during the epidemiological investigation of *C. burnetii* prevalence in ticks [55], while *C. burnetii* detection assays based only on *IS1111* may lead to misidentification with *Coxiella*-like endosymbionts [56]. Reeves et al. 2006 were able to amplify a 612 bp *icd* fragment, displaying 93.0% homology with *C. burnetii*, from a *Coxiella*-like bacterium that infects ticks [57]. In contrast, Elsa et al., 2015, proved that *icd* was not amplified from the endosymbiont of *Ornithodoros capensis* ticks and highlights the fact that the amplification of a specific genetic marker strongly depends on the PCR method and the primer sequences used [56].

In this study, we used a combination of primers targeting different markers, *IS1111* and *icd*, to guarantee the specificity of *C. burnetii* detection.

Standardizing a methodology for the detection of *C. burnetii* DNA across laboratories is essential to make sure that no cross-reaction exists not only with other abortive agents but also with *Coxiella*-like organisms.

## 5. Conclusions

The findings of the present study for the detection of *C. burnetii* infection in small ruminants in different geographical regions of Pakistan indicates that coxiellosis is endemic in this country. Since there is very limited awareness of the disease among veterinarian and farm workers of the studied livestock farms, an awareness-raising campaign should be launched to educate livestock farmers and professionals about proper preventive and control measures for coxiellosis.

This survey highlights the importance of clinical herd health management to avoid economical losses in small ruminants.

## Figures and Tables

**Table 1:** Seroprevalence of coxiellosis in small ruminants in seven districts of Punjab, Pakistan.

Location	Positive/Tested	Prevalence, %	95% CI	OR ^#^	95% CI *	Statistics
Layyah	74/283	26.2	21.1–31.7	5.79	1.75–19.11	χ^2^ = 49.689 df = 6 *p* = 0.000
Bhakkar	37/208	17.8	12.8–23.7	3.54	1.04–11.9	
Khushab	6/45	13.3	5.1–26.8	2.52	0.59–10.69	
Okara	15/132	11.4	6.5–18.0	2.1	0.58–7.56	
Bhawalpur	4/37	10.8	3.0–25.4	1.98	0.42–9.43	
Khanewal	14/243	5.8	3.2–9.5	1	0.28–3.61	
Rajanpur	3/52	5.8	1.2–15.9	1	-	
Overall	153/1000	15.3	12.3–18.7	-	-	

Individual prevalence was significantly different in the farms investigated, χ^2^ = 49.689, df = 6, *p* = 0.000. ^#^ OR (odds ratio); * CI (confidence interval).

**Table 2:** Analysis for the serological results of coxiellosis in small ruminants in seven farms in the Punjab, Pakistan, in relation to potential risk factors.

Variable	Category	Pos./Tested	Prev. %	95% CI	OR	95% CI	*p*-Value
Species	Sheep	78/500	15.6	12.5–19.1	1.047	0.74–1.47	χ^2^ = 0.069 df = 1 *p* = 0.792
	Goat	75/500	15.0	12.0–18.4	1	-	
Age	Up to 1	22/133	16.5	10.0–23.1	1.082	0.63–1.85	χ^2^ = 6.603 df = 3 *p* = 0.085
	>1–2.5	20/186	10.8	6.1–15.6	1	-	
	>2.5–4	47/241	19.5	14.9–25.1	1.406	0.93–2.12	
	>4	64/440	14.5	11.6–18.4	0.653	0.38–1.14	
Sex	Male	20/107	18.7	11.8–27.4	1.314	0.78–2.21	χ^2^ = 1.064 df = 1 *p* = 0.302
	Female	133/893	14.9	12.6–17.4	1	-	
Parity	Nulliparous	29/198	14.6	11.7–20.2	1.203	0.63–2.29	χ^2^ = 0.353 df = 2 *p* = 0.838
	Multiparous	82/582	14.1	12.7–18.7	1.191	0.65–2.18	
	Primiparous	22/113	19.4	7.5–21.4	1	-	
Lactation	Lactating	34/399	8.5	16.8–24.0	2.800	1.84–4.25	χ^2^ = 23.1064 df = 1 *p* = 0.000
	Non-lactating	99/494	20.0	5.8–11.4	1	-	
Reproductive Status	Pregnant	43/320	10.6	14.2–20.6	1.747	1.15–2.65	χ^2^ = 10.483 df = 1 *p* = 0.002
	Non-pregnant	99/573	17.3	7.5–14.6	1	-	
Rep. Disorder	Abortion	48/93	51.6	41.0–62.1	10.174	6.37–16.26	χ^2^ = 133.984 df = 4 *p* = 0.000
	Premature delivery	12/37	32.4	18.0–49.8	4.578	2.21–9.47	
	Stillbirth	14/44	31.8	18.6–47.6	4.451	2.26–8.75	
	Repeat breeding	1/4	25.0	0.6–80.6	3.179	0.33–30.93	
	No	78/822	9.5	7.6–11.7	1	-	
Body Condition	Weak	32/70	45.7	33.7–58.1	7.984	4.73–13.49	χ^2^ = 124.868 df = 3 *p* = 0.000
	Does with weak kid delivery	22/49	44.9	30.7–59.8	7.725	4.20–14.20	
	Ewes with weak lamb delivery	20/53	37.7	24.8–52.1	5.746	3.15–10.49	
	Apparently good	79/828	9.5	7.6–11.7	1	-	

**Table 3:** Detection of *Coxiella burnetii* DNA in 29 pooled serum samples.

Serum Pools	Positive/Tested	Prevalence, %	95% CI	OR	95% CI	Statistics
Sheep	2/15	13.3	1.7–40.5	1	-	χ^2^ = 0.333 df = 1 *p* = 0.564
Goat	3/14	21.4	4.7–50.8	1.61	0.10–4.05	
Overall	5/29	17.2	6.60–34.2	-	-	

**Table 4:** Prevalence of *C. burnetii* DNA in 55 pools of ticks collected from small ruminants.

Tick Pools	Positive/Tested	Prevalence %	95% CI	OR	95% CI	Statistics
Sheep	9/29	31.0	15.3–50.8	4.03	0.05–1.22	χ^2^ = 4.668 df = 1 *p* = 0.031
Goat	2/26	7.7	0.9–25.1	1	-	
Overall	11/55	20.0	10.99–32.10	-	-	
